# Glaucoma and Myopia: Diagnostic Challenges

**DOI:** 10.3390/biom13030562

**Published:** 2023-03-20

**Authors:** Michelle T. Sun, Matthew Tran, Kuldev Singh, Robert Chang, Huaizhou Wang, Yang Sun

**Affiliations:** 1 Department of Ophthalmology, School of Medicine, Stanford University, Palo Alto, CA 94305, USA; 2 School of Medicine, University of Nevada, Reno, NV 89557, USA; 3 Beijing Tongren Eye Center, Beijing Tongren Hospital, Capital Medical University, Beijing 100730, China; 4 Palo Alto Veterans Administration, Palo Alto, CA 94304, USA

**Keywords:** glaucoma, myopia, high myopia, primary open angle glaucoma, intraocular pressure, IOP, POAG, review

## Abstract

The rising global prevalence of myopia is a growing concern for clinicians, as it predisposes patients to severe ocular pathologies including glaucoma. High myopia can be associated with clinical features that resemble glaucomatous damage, which make an accurate glaucoma diagnosis challenging, particularly among patients with normal intraocular pressures. These patients may also present with established visual field defects which can mimic glaucoma, and standard imaging technology is less useful in disease detection and monitoring due to the lack of normative data for these anatomically unique eyes. Progression over time remains the most critical factor in facilitating the detection of early glaucomatous changes, and thus careful longitudinal follow-up of high-risk myopic patients is the most important aspect of management. Here, we review our current understanding of the complex relationship between myopia and glaucoma, and the diagnostic challenges and limitations of current testing protocols including visual field, intraocular pressure, and imaging. Furthermore, we discuss the clinical findings of two highly myopic patients with suspected glaucoma.

## 1. Introduction

Myopia has become increasingly prevalent worldwide, reflecting the significant changes in lifestyle behaviors and predominant indoor near work among the younger population, particularly East Asians [1,2]. During the past few decades, there has been increased recognition of trends towards the high and significantly increased prevalence of myopia in schoolchildren in many areas of the world including Finland, Singapore, the United States, and Hong Kong during the course of the 20th century [3,4,5,6]. A meta-analysis of 145 published studies involving 2.1 million participants estimated that myopia (spherical equivalent refraction of −0.50 diopters (D) or worse) affected approximately 22% of the world population in 2000, and is projected to affect 50% of the world population by 2050 [1]. Furthermore, in the developed countries of East and Southeast Asia, the prevalence of myopia among high school-aged children has now reached 80–90% [7,8]. The rising global prevalence of myopia is a growing concern for clinicians, as it predisposes patients to severe ocular pathologies including glaucoma.

The association between myopia and primary open angle glaucoma (POAG) is well established [9,10,11,12]. Numerous large population-based studies have demonstrated an increase in POAG prevalence with increasing myopia, and that this association is more pronounced for higher degrees of myopia [13,14,15,16,17]. For example, The Blue Mountains Eye Study found that low myopes (−1.0 D to −3.0 D) had a two-fold increased risk of glaucoma compared to non-myopes, while moderate-to-high myopes (−3.0 D or worse) had a three-fold increased risk [14]. Additionally, The Los Angeles Latino Eye Study and Singapore Malay Eye Study both measured axial length, and found that an increasing axial myopia was associated with a higher risk of glaucoma [15,16]. However conflicting evidence does exist, particularly around the range of refractive errors considered important for glaucoma risk. One comparative study found no significant differences in the degree of glaucomatous optic neuropathy between those with mild-to-moderate myopia (less than −8.0 D) and non-myopic patients, while the Ocular Hypertensive Treatment Study found no association with POAG for any degree of myopia [18,19]. Similarly, a smaller Chinese study found that axial eye length showed no association with visual field loss [20]. However, a more recent meta-analysis of 24 studies from 11 countries found evidence for a dose-response relationship between the degree of myopia and POAG [17]. Significant heterogeneity existed amongst studies reporting the risk estimates of POAG for low myopia (less than −3.0 D), and once outliers were excluded, the pooled odds ratio for low myopia was 1.77 (95% CI 1.41–2.23) and 2.46 (95% CI 1.93–2.15) for high myopia. Furthermore, for each 1.0 D increase in myopia, the risk of glaucoma increased by approximately 20%, but with a steeper increase for high myopia, suggesting a non-linear relationship [17]. Further longitudinal studies will be of value to better understand the complex relationship between myopia and glaucoma.

## 2. Anatomical Basis for Disease and Corresponding Clinical Findings

The mechanisms for the relationship between myopia and glaucoma are incompletely understood. The mechanical hypothesis relates to the differing anatomy of myopic eyes, which have longer axial lengths and thinner sclera [21]. These changes result in the deformity of the lamina cribrosa, thought to be related to stress-related mechanical changes due to higher scleral tension across the lamina cribrosa, contributing to a higher susceptibility to glaucomatous optic neuropathy [11,22,23]. A study of Korean children found that those with myopic shift demonstrated a progressive optic nerve head tilt, suggesting this clinical finding is acquired and likely arises from mechanical scleral stretching [24]. Assuming that retinal ganglion cells are non-distensible, any lengthening of these fibers associated with axial elongation leads to mechanical strain and predisposition to dysfunction [25]. Imaging of myopic discs using swept-source optical coherence tomography (OCT) have been able to demonstrate a posterior deformation in Bruch’s membrane with correlation to functional glaucomatous damage, while retinal ganglion cell axon compaction in optic discs with increasing myopia has been shown using confocal scanning ophthalmoscopy [26,27].

Peripapillary anatomical changes are also likely to play a role in the increased susceptibility for glaucomatous optic neuropathy in high myopia [28,29]. Beta-zone peripapillary atrophy is one of several established morphologic features of glaucomatous optic neuropathy, and the progression of beta-peripapillary atrophy has been found to correlate with myopia progression and axial length elongation in children [30,31,32]. Beta-peripapillary atrophy has been associated with a higher risk of glaucoma progression, and is more pronounced in patients with high myopia-related glaucoma [33,34]. The peripapillary delta zone is also a particular area of interest in myopic eyes, and represents the peripapillary scleral flange, defined as the region between the peripapillary ring and merging of the optic nerve dura mater with the posterior sclera [28,35,36]. The axial elongation associated with glaucoma has been found to correlate with the peripapillary delta zone, with such patients having a more elongated peripapillary scleral flange and larger optic discs [37]. As Bruch’s membrane thickness is independent of axial length, the opening of Bruch’s membrane at the optic nerve head is shifted temporally in high myopia, with a resultant lack of Bruch’s membrane at the temporal disc and histological equivalent of a gamma zone [38].

The axial elongation-induced optic disc tilt and subsequent stretching of the temporal peripapillary scleral flange results in characteristic changes to the optic nerve head, which, when affected by glaucoma, have important differences to non-myopic glaucomatous optic nerves. Myopic discs are larger and can have a greater disc area [39]. In addition to a vertically elongated and tilted shape, obscuration of the temporal and/or nasal rims and increased beta-peripapillary atrophy, myopic glaucomatous discs tend to have more shallow diffuse cupping which can be difficult to objectively quantify [34]. Importantly, the features of myopic and glaucomatous changes to the optic nerve head can overlap, and thus consideration of the entire clinical picture is critical in making an accurate diagnosis.

## 3. Diagnostic Challenges

### 3.1. Visual Field

Differentiating myopia-related optic neuropathy and normal tension glaucoma (NTG) can be very challenging due to the constellation of clinical findings particularly associated with high myopia. It has been well recognized that myopic patients with normal intraocular pressures (IOPs) can present with glaucomatous visual field defects, which can be attributed to myopia rather than to glaucoma [40,41,42,43]. Of the 894 unique visual fields from the Zhongshan Ophthalmic Center–Brian Holden Vision Institute High Myopia Registry Study classified at baseline, 16% of these in young high myopes were found to mimic classic glaucomatous defects and follow-up is ongoing to determine which proportion of these progress [41]. A small series of 16 young myopic Chinese patients previously diagnosed with glaucoma or glaucoma suspects were followed for 7 years and found to have non-progressive visual field and optic disc cupping irrespective of IOP-lowering therapy [40]. These studies highlight the fact that a proportion of myopic patients may be misdiagnosed as having glaucoma and be taking unnecessary treatment, and thus the ability to distinguish the two is important, but often not straightforward.

Given the many different types of visual field defects associated with the myopia described, most recently, a proposed classification system for highly myopic eyes without glaucoma or pathological changes was suggested as a way to standardize reporting [42]. Defects described included glaucoma-like (paracentral defects, nasal step, paracentral arcuate and arcuate), myopia-like (enlarged blind spot, vertical step, partial peripheral rim and non-specific) and combined, with approximately 10% of eyes demonstrating the glaucoma-like defects which tended to be associated with longer axial length [42]. Progression over time and age at progression both represent important factors in differentiating glaucoma-like visual field defects from true glaucoma [40,44]. Furthermore, the nature of progression may also be important with previous suggestions that myopia may result in regional susceptibility of the nerve to damage, and thus extension of a field defect in a region which corresponds anatomically to tilt may not be as significant as a defect in a region corresponding to an optic nerve that previously appeared healthy [45].

### 3.2. Intraocular Pressure

The role of IOP in myopic glaucoma patients can present both diagnostic and management challenges. In numerous previous population-based studies, NTG has been found to comprise the majority of open-angle glaucoma (OAG) in Asians, with rates ranging from 52–92% [46,47,48,49,50,51,52]. Interestingly, a prior study found that glaucoma was associated with elevated IOP in myopic eyes less than 27.5 mm, whilst in longer eyes, factors not including IOP (larger optic disc, longer axial elongation and older age) were associated with glaucoma [53]. This may reflect the increased susceptibility of more highly myopic eyes to glaucomatous damage at lower IOPs, given the anatomical differences described previously. The higher prevalence of high myopia in Asians may thus be partially accounting for the higher prevalence of NTG in Asian populations.

Recent genetic research has also implicated IOP as a key mediator in the causal pathway between myopia and POAG [54]. Mendelian randomization (MR) analyses have found bidirectional genetic causal associations between myopia and POAG as well as IOP, suggesting the possibility of a converse directional relationship [54]. Additionally, multivariate genomic structural modeling suggests that the causal effect of myopia on POAG is predominantly mediated by IOP [54]. Other MR analyses have also reported genetic correlations between POAG and myopia [54,55]. These associations, however, did not reach statistical significance in Asian eyes, suggesting that myopia-related glaucoma in Asian eyes may also involve IOP-independent mechanisms such as ethnic differences in blood pressure variability.

There is evidence to suggest that baseline IOP, and IOP diurnal patterns may differ between myopes and non-myopes. Young myopic adults without glaucoma have been found to have higher daytime IOPs compared to those without myopia, as well as higher nocturnal supine IOP elevation [56,57,58,59]. In patients with high-tension POAG, there was greater IOP fluctuation after exercise in high myopes as compared to controls, while differences in the 24 h IOP range and nocturnal IOP elevation were found to differ in myopic OAG patients compared to those without myopia [60,61]. These differences may be attributed in part, or a combination of variations in choroidal thickness, choroidal vascular differences and/or scleral rigidity found in highly myopic eyes [62,63]. These differences present unique challenges to clinicians faced with interpreting and managing the IOP of myopic glaucoma patients and those with suspected glaucoma. The concept of a target IOP in a myopic patient may be more difficult, particularly in the context of a patient presenting with normal or low IOPs. Caution should be taken when targeting an aggressively low IOP in the absence of established progression, given the significantly higher risk of hypotony maculopathy following filtration surgery in myopes [64].

### 3.3. Imaging

In addition to glaucomatous-like visual field defects, optic nerve morphology of myopes and particularly of high myopes, is often abnormal and thus difficult to assess objectively using currently available imaging technology. Myopes are known to have a decreased retinal nerve fiber layer (RNFL) at baseline and RNFL bundles can be shifted temporally, resulting in thicker temporal quadrants and nasal thinning, which can be misleading if compared to the inbuilt normative database within which moderate–high myopic patients are not well represented [65,66,67]. Furthermore, the anatomical differences and peripapillary changes can make the optic disc segmentation challenging, and thus increase the likelihood of artefacts or erroneous RNFL measurements, which should be interpreted with caution [68]. Careful visual inspection of the circumpapillary image can allow a clinician to assess the quality of the scan, as well as quality of the RNFL segmentation, and paying close attention to the inner circle scan report can be helpful tools to accurately interpret RNFL scans from myopic patients [69,70].

The ability to detect myopia-associated glaucoma-like optic neuropathy has also been improved by advances in swept-source OCT (SS-OCT) and OCT angiography (OCTA) [71,72,73]. Imaging studies have shown that SS-OCT can visualize the entire layer of the choroid and sclera in patients with high myopia [71,74]. Using SS-OCT, the angle of scleral bending has been correlated with visual defect severity in highly myopic eyes [71]. OCTA has also emerged as a useful vascular imaging modality to detect early glaucomatous nerve damage, which has been associated with focal defects in peripapillary retinal perfusion around the optic nerve [73,75]. By characterizing the features of glaucoma in myopic eyes using SS-OCT and OCTA, artificial intelligence (AI) has recently been applied to large databases of fundus photographs to develop algorithms which have shown promise as a potential tool for detecting glaucoma in populations with a high incidence of myopia [73,76].

Several previous studies have demonstrated that the ganglion cell complex (GCC) parameters are superior to RNFL at detecting glaucoma in the context of high myopia [77,78,79,80,81]. Vertical scans of the macula have also been shown to be useful for evaluating RNFL thickness in myopic eyes [82]. Whilst RNFL has been found to correlate with refractive error, GCC was found to have no significant relationship with refractive error, and furthermore may better agree with visual field defects as compared to RNFL [80,81]. While the results of a meta-analysis found no significant differences between the two parameters, a more recent longitudinal study found that highly myopic eyes with progressive macula ganglion–inner plexiform layer thinning had a significantly higher risk of developing visual field defects, while there was no correlation with RNFL thinning, in contrast to non-myopes [83,84]. Although there is ongoing uncertainty regarding the optimal utilization of various OCT parameters high myopes, the current literature suggests that monitoring the GCC longitudinally may be particularly important in these patients; although macular disease, which is reported to be found in up to 10% of European and 28% of Asian high myopes, can distort ganglion cell analyses and render these readings less reliable [85,86]. There remains an unmet need to develop a normative database of myopic patients with which to compare patients with established or suspected glaucoma to.

## 4. Case Discussion

The two cases below demonstrate the spectrum of disease seen in myopic patients also suspected to have glaucoma. In addition to history, examination and investigations, adequate follow-up over time is the most critical factor in ensuring the correct diagnosis and management.

### 4.1. Case 1

**Figure 1:**
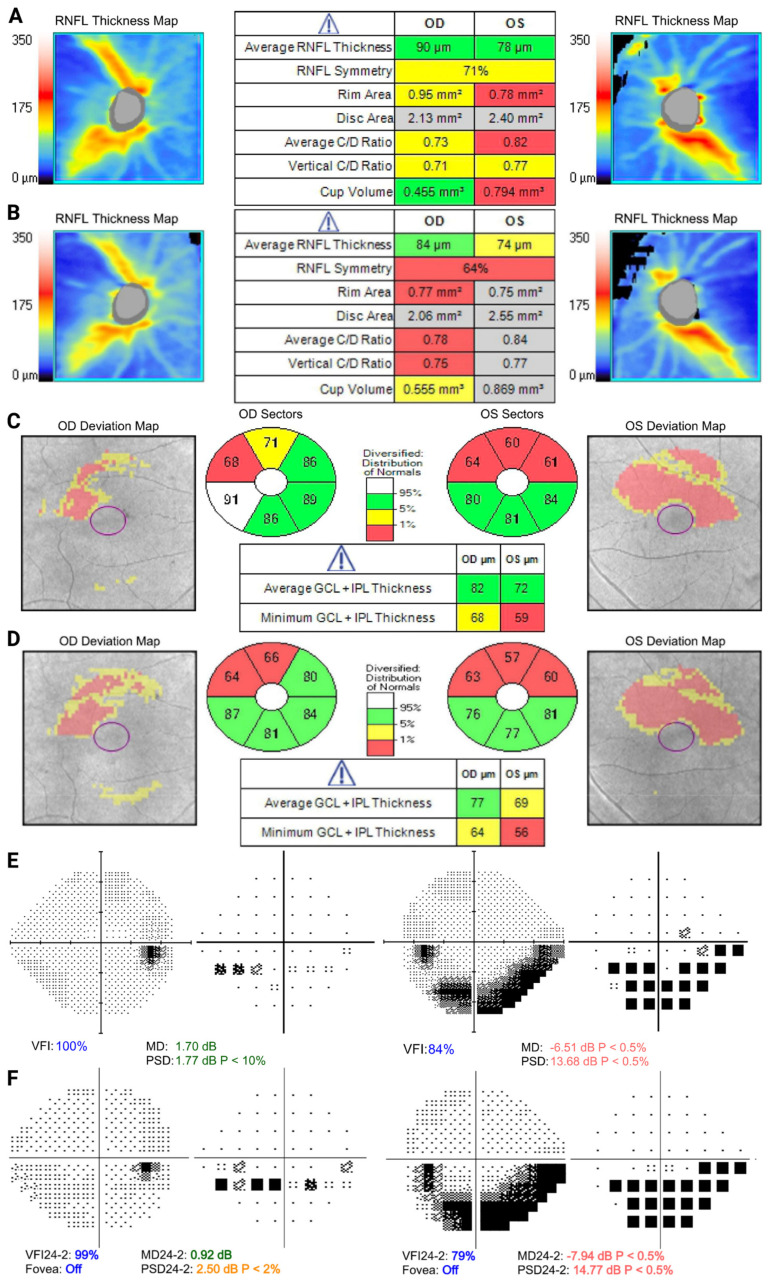
Optical coherence tomography (OCT) and visual field examination of patient 1 at baseline (**top**) and 5-year follow-up (**bottom**). (**A**) Retinal nerve fiber layer demonstrates stable mild right inferior thinning and more pronounced left superior thinning, with progression more pronounced in the left eye over time (**B**). (**C**,**D**) Bilateral superior–temporal ganglion–cell complex defects, worse in the left eye. (**E**) Visual field shows a mild early inferior nasal arcuate defect in the right eye and a denser inferior arcuate in the left eye, which progressed over time (**F**).

A 59-year-old Asian male with a myopic prescription of −8.0 D OD and −7.0 D OS presented with evidence of progression in his visual field defects over five years of follow-up. He had a diagnosis of NTG, and his IOPs had been stable in the mid-teens for several years on topical medications. Optic disc examination revealed slightly tilted, cupped discs with mild peripapillary atrophy. OCT RNFL demonstrated stable mild right inferior thinning, and more pronounced left superior thinning, with bilateral superior–temporal ganglion–cell complex defects (Figure 1A). The visual field showed a mild early inferior nasal arcuate defect in the right eye, and a denser inferior arcuate in the left, both of which had progressed gradually over time (Figure 1B,C). These changes are all in keeping with glaucomatous optic neuropathy, with myopia being a pre-disposing factor. Given the proximity to fixation, and evidence of progression, this patient was recommended further therapy to lower IOP, with a target likely in the single digits.

### 4.2. Case 2

**Figure 2:**
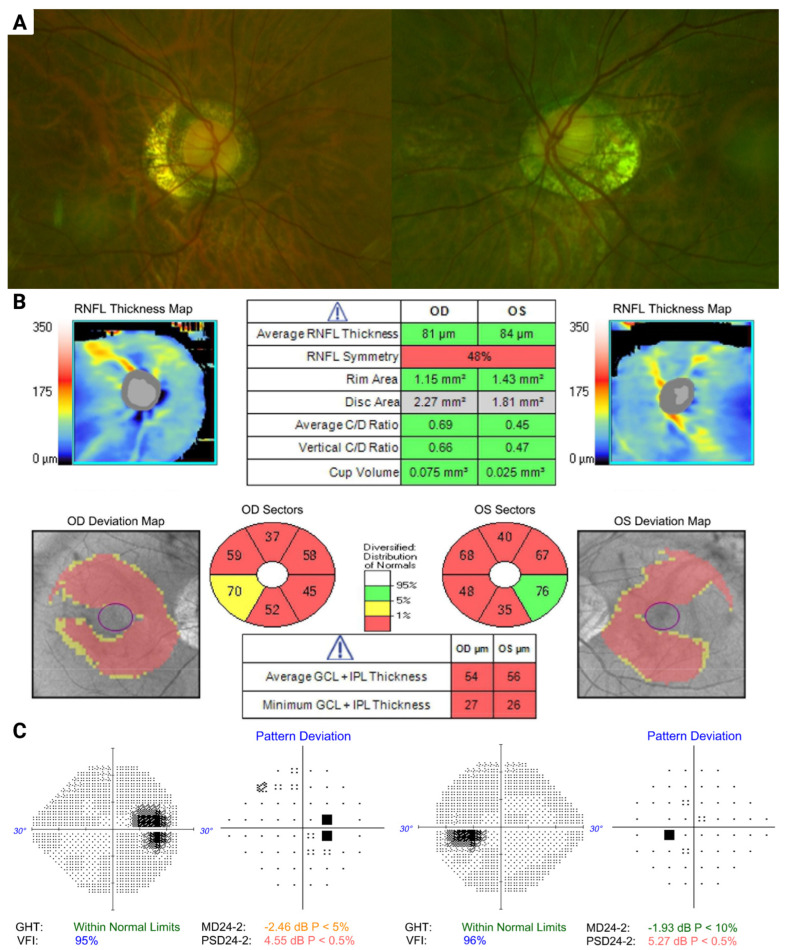
Color fundus imaging, optical coherence tomography (OCT) and visual field examination of patient 2. (**A**) Optic nerves are tilted and ovoid in shape, with pronounced peripapillary atrophy. (**B**) OCT demonstrates inferior retinal nerve fiber layer (RNFL) thinning and bilateral diffuse ganglion cell complex (GCC) thinning with staphylomatous changes. (**C**) Visual field shows bilateral enlarged blind spots.

A 50-year-old female Asian with a myopic prescription of −15.75 D OD and −15.0 D OS presented for a review of visual field defects noted at the baseline examination five years prior. She had been followed as a glaucoma suspect/ocular hypertensive, with IOP at 20 OU on latanoprost in both eyes. Her optic nerves were tilted and ovoid in shape, with pronounced peripapillary atrophy, worse in the left eye (Figure 2A). OCT demonstrated inferior RNFL thinning and bilateral diffuse GCC thinning but was confounded by an artefact due to the staphylomatous changes (Figure 2B). Her visual field demonstrated bilateral enlarged blind spots, which had remained stable over time (Figure 2C). Given the stability of the visual fields, this patient remains under observation as a suspect and has not required any escalation of treatment.

## 5. Conclusions

Our understanding of the complex relationship between myopia and glaucoma continues to develop, particularly with the increasing prevalence of myopia worldwide. Myopia is an established risk factor for OAG, but often can present with normal IOP which complicates the diagnosis and management. Myopia, in particular axial high myopia, is associated with anatomical changes with resultant characteristic differences in the clinical appearance of the optic nerve head and peripapillary region, making the assessment of optic nerve cupping especially challenging. These patients often present with established visual field defects which can mimic glaucoma, and standard imaging technology is less useful in disease detection and monitoring due to the lack of normative data for these anatomically unique eyes. Progression over time remains the most critical factor, and thus careful longitudinal follow-up of these patients is the most important aspect of management. As our technology continues to improve, there will no doubt be further advances to aid the detection and management of patients with myopia and glaucoma.

## Data Availability

Not applicable.
